# Delays in the Management of Retroperitoneal Sarcomas

**DOI:** 10.1155/2010/702573

**Published:** 2010-10-26

**Authors:** Jojanneke Seinen, Martin Almquist, Emelie Styring, Anders Rydholm, Mef Nilbert

**Affiliations:** ^1^Departments of Oncology, Institute of Clinical Sciences, Skåne University Hospital, Lund University, 22185 Lund, Sweden; ^2^Departments of Surgery, Institute of Clinical Sciences, Skåne University Hospital, Lund University, 22185 Lund, Sweden; ^3^Departments of Orthopedics, Institute of Clinical Sciences, Skåne University Hospital, Lund University, 22185 Lund, Sweden; ^4^Department of Surgical Oncology, University Medical Centre Groningen, University of Groningen, 9700 Groningen, The Netherlands; ^5^Clinical Research Centre, Copenhagen University, Hvidovre University Hospital, 2650 Hvidovre, Denmark

## Abstract

Retroperitoneal sarcomas are rare and treatment should optimally be centralized. Despite successful centralization with 90% of the patients referred prior to surgery, delays occur, which led us to assess lead times in a population-based series. *Method*. Patients diagnosed with retroperitoneal sarcoma in the southern Sweden health care region 2003–2009 were eligible for the study. Data on referrals and diagnostic investigations were collected from clinical files from primary health care, local hospitals, and from the sarcoma centre. Lead times were divided into patient delays and health care delays caused by primary health care, local hospitals, or procedures at the sarcoma centre. *Results*. Complete data were available from 33 patients and demonstrated a median patient delay of 23 days (0–17 months) and median health care delay of 94 days (1–40 months) with delays of median 15 days at the general practitioner, 36 days at local hospitals, and 55 days at the sarcoma centre. *Conclusion*. Centralization per se is not sufficient for optimized and efficient management. Our findings suggest that delays can be minimized by direct referral of patients from primary health care to sarcoma centers and indicate that development of coordinated diagnostic packages could shorten delays at the sarcoma centre.

## 1. Introduction

Retroperitoneal sarcomas represent 0.1% of all malignancies and are often clinically challenging due to anatomical proximity to vital structures and a considerable risk for local recurrence [1]. The rarity, complex diagnostics, and surgical challenges imply that these tumors should be managed by experienced sarcoma teams. Centralized treatment per se may not be sufficient since delays that may allow tumor progression, complicate surgery, increase the risk of local recurrence, and cause unnecessary worry for patients are experienced at general practitioners and local hospitals as well as at sarcoma centers [2–6]. Delays have been linked to adverse outcome in several tumor types, including breast cancer, colorectal cancer, urothelial cancer, and esophageal cancer [7–10]. Benchmarks for timely management have not been defined in retroperitoneal sarcoma, but large tumor size represents an adverse prognostic factor, which strongly argues for efficient management. Detailed understanding of the causes of delays is needed for optimized management, which led us to identify diagnostic lead times related to the patient, general practitioners, and procedures at local hospitals and at the sarcoma centre in a population-based series of retroperitoneal sarcoma patients.

## 2. Materials and Methods

Primary, histologically verified retroperitoneal sarcoma was, in the southern Sweden health care region (1.5 million inhabitants), diagnosed in 39 patients between 2003 and 2009. Complete data were available from 33 patients. All relevant medical records from general practitioners, local hospitals and the sarcoma centre were collected. Patient's delay was defined as the time from onset of self-reported symptoms to the first visit to a medical professional, which could be a general practitioner or a specialist. Health care delay was defined as the time from the first visit to the start of treatment, which was in most cases surgery. Hereunder, lead times were specified to occur in primary health care (from the first visit to a general practitioner until referral to a local hospital or sarcoma centre), at local hospitals (from the first visit until the start of treatment or referral to the sarcoma centre) or in the sarcoma centre (from the first visit until start of treatment). Pathology lead time was defined as the time from referral for cytology/biopsy until confirmed malignancy. Radiology lead time was defined as the time from referral for the first investigation to the result of the final investigation. All lead times were expressed as median times in order to minimize the impact of skewed distributions. According to Swedish health care regulations, the study represents a quality control project, for which ethical permission is not required.

## 3. Results

**Figure 1:**
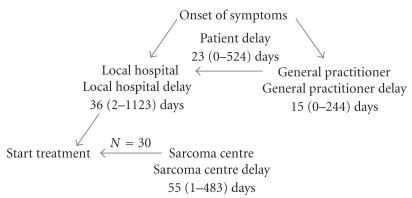
Overview of the different median lead times.

**Figure 2:**
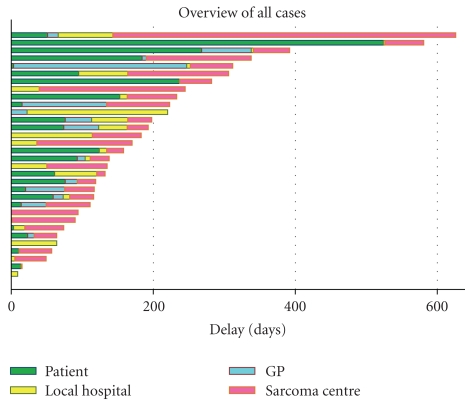
Bar chart demonstrating individual patients' lead times (one outlier with a 3-year local hospital delay was omitted for reasons of illustration).

Complete data were available from 33 patients (Table 1). The mean age at diagnosis was 66 (21–87) years, and the study included 17 men. Liposarcoma and leiomyosarcoma were the predominant histopathological subtypes. The majority (*n* = 19) of the tumors were high grade and the mean tumor size was 21 (4–60) cm. Median and individual delays are presented in Figures 1 and 2. The median lead time from onset of self-reported symptoms to the first medical visit was 23 days (0–17 months). Though 15 patients consulted a medical professional within 1 month of onset of symptoms, patient's delay was the predominant in 12/33 cases. The most common symptoms (*n* = 14) were pain or abdominal discomfort whereas 4 tumors were incidentally diagnosed at surgery or radiologic investigations for other causes. The total health care lead time was median 94 days (1–40 months) and consisted of a general practitioner's lead time of median 15 days (0–8 months), a local hospital lead time of 36 days (0–37 months), and a sarcoma centre lead time of 55 days (1–16 months). The longest delays were caused by erroneous primary diagnosis (7, 16, and 40 months) and comorbidity that required complimentary medical procedures prior to surgery (5, 8, and 19 months). Among the 17 patients who consulted a general practitioner, 11 were referred within 1 month of the first visit and 6 were referred directly to the sarcoma centre. From local hospitals, 11/23 patients were referred to the sarcoma centre within 1 month, which implies that half of the patients spent more than a month at this stage. The sarcoma centre lead time of median 55 days represented the longest delay in 12/33 patients. 

**Figure 3:**
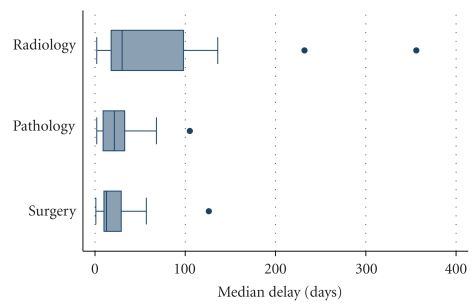
Box-plot demonstrating the radiology, pathology, and surgery lead times at the sarcoma centre. Outliers are marked by •.

The diagnostic delays were divided into pathology and radiology lead times (Figure 3). In 25 patients, a fine needle aspiration cytology and/or core needle biopsy was performed, 14 of which were performed at the sarcoma centre. The pathology lead time was median 22 days (0–4 months) with some of the longest delays caused by inconclusive results from cytology/histopathology. Repeated needle biopsies were required in 5 patients, and 11 patients were operated on without a histologically confirmed diagnosis. Radiology lead time was median 36 days (0–8 months) and the investigations included abdominal CT scans in all but one patients, complemented with CT scans of the thorax and renography in most patients. The delay from completed diagnostics to surgery was median 13 (1–57) days.

## 4. Discussion

Management of retroperitoneal sarcomas requires a multidisciplinary approach with contributions from radiologists, pathologists, surgeons, and oncologists. Though primary surgery at a sarcoma centre is beneficial, efficient diagnostics is central. In southern Sweden, centralized treatment has been promoted since a decade with 90% of the patients currently referred to the sarcoma centre before surgery. In order to characterize delays and causes hereof, we assessed lead times in our population-based cohort. The median patient's delay was only 3 weeks, though considerable longer delays occurred in some cases and indeed represented the predominant delay in almost half of the patients (Figure 2). It should, however, be kept in mind that these data are based on self-reported symptoms and thus prone to bias compared to the other lead times, which are based on documented dates of referral. No comparison can be made to published delays for retroperitoneal sarcoma, but in soft-tissue sarcomas, considerable delays have been reported. Brouns et al. reported median patient's delays of 2 months in more than half of the patients and of at least 6 months in 20% of patients [11]. Clark and Thomas reported lead times of median 12 months in referring sarcoma patients from general practitioners [12]. We found a median general practitioners' delay of 16 days, which indeed represents the shortest health care lead time. Considering the rarity of retroperitoneal sarcomas, these prompt reactions to suspected malignancy are impressive. 

Our data demonstrate that the time is lost at the subsequent step for patients that are primarily referred to a local hospital. The median lead time from the local hospital to the sarcoma centre was 5 weeks, and in several cases investigations originally performed at the local hospital were repeated at the sarcoma centre. This observation strongly suggest that patients with suspected retroperitoneal sarcoma should be directly referred to the sarcoma centre in order to avoid unnecessary procedures and reduce lead times. 

A series of investigations are typically needed in the diagnostic workup and surgical planning of retroperitoneal sarcoma and treatment decisions are made at multidisciplinary conferences. Against this background, it is not surprising that the sarcoma centre lead time of median 8 weeks was predominant. Additional morphological investigations needed to reach a pathological diagnosis and requests for complimentary imaging were identified as the major causes of delay (Figure 3). Radiology delays were the predominant with median delays exceeding 5 weeks in half of the cases. Separate requests for different examinations rather than coordinated examinations likely contributed to the delays. Due to limited resources at the sarcoma centre, a significant number of radiology investigations were also performed at local hospitals, which contributed to longer lead times, since the results of the investigations were not immediately available to the surgeons at the sarcoma centre. This leads us to suggest that retroperitoneal sarcoma radiology packages could be defined to achieve efficient and coordinated radiologic investigations and hereby reduce lead times. The pathology lead time of median 3 weeks leaves room for improvement. We suggest that cytology specimens from fine needle examinations should be immediately evaluated. Hereby, representative material can be directly ensured and direct resampling ordered when necessary. Finally, the median lead time of 2 weeks from complete diagnostic workup until surgery is considered acceptable against the background of coordinated efforts from oncological surgeons. 

Studies that have addressed the diagnostic delays in other less common tumor types; that is, esophageal cancer and cancer of the urinary tract have reached results similar to ours. Esophageal cancer also requires extensive diagnostic workup followed by complex surgery when possible. Grotenhuis et al. showed a median hospital delay of 7 weeks from the endoscopy at which a diagnostic biopsy was obtained until surgery and median 2 weeks from the treatment decision at a multidisciplinary conference until surgery [10]. The authors could also demonstrate that rapid management was associated with favorable outcome as regards both morbidity and mortality. Holmäng and Johansson analyzed diagnostic and treatment delays in patients with upper urothelial cancer with a median delay from urography to surgery of 3–8 weeks with considerable differences in delay and tumor stage between different hospitals [9]. They suggest that large tumors lead to more rapid workup and earlier surgery. The rarity and variable clinical course of retroperitoneal sarcoma precludes analysis of outcome, but interestingly, some of the longest doctor's delays occurred in patients with large tumors.

We conclude that a substantial number of patients in this population-based retroperitoneal sarcoma cohort experience considerable diagnostic delays. General practitioners' delays were acceptable, local hospital delays should be possible to minimize, and sarcoma center delays could be shortened through improved coordination. Our data point to three possible improvements. Patients with suspected retroperitoneal sarcomas should be directly referred to sarcoma centers to reduce lead times at local hospitals. At the sarcoma centre, radiologic and pathologic investigations should be coordinated, for example, through predefined radiology packages and prioritized evaluation of cytology/pathology specimens. Finally, lead times should be prospectively registered in order to map bottle necks in different systems and evaluate the effect of altered routines for diagnostic workup. Such data would also allow for establishment of clinical diagnostic guidelines and limits for timely care of retroperitoneal sarcoma.

## Figures and Tables

**Table 1:** Summary of clinicopathologic characteristics.

Characteristics	*N* (%)
Sex (male : female)	17 : 16
Age, mean (range)	66 (21–87)
Tumor size, cm, mean (range)	21 (4–60)
Histopathologic type	
Liposarcoma	13 (40)
Leiomyosarcoma	8 (24)
Spindle cell sarcoma	4 (12)
Inflammatory myofibroblastic sarcoma	1 (3)
GIST	1 (3)
Carcinosarcoma	1 (3)
Atypical solitary fibrous tumor	1 (3)
NOS	4 (12)
Malignancy grade	
Low	9 (27)
Intermediate	1 (3)
High	19 (58)
NOS	4 (12)

GIST: gastrointestinal stromal cell tumor.

NOS: not otherwise specified.
